# The effect of the Lokomat^®^ robotic-orthosis system on lower extremity rehabilitation in patients with stroke: a systematic review and meta-analysis

**DOI:** 10.3389/fneur.2023.1260652

**Published:** 2023-12-06

**Authors:** Lina Wu, Gui Xu, Qiaofeng Wu

**Affiliations:** Department of Rehabilitation, Foresea Life Insurance Nanning Hospital, Nanning, Guangxi Province, China

**Keywords:** Lokomat^®^, stroke, lower extremity function, rehabilitation, meta-analysis

## Abstract

**Background:**

The Lokomat^®^ is a device utilized for gait training in post-stroke patients. Through a systematic review, the objective was to determine whether robot-assisted gait training with the Lokomat^®^ is more effective in enhancing lower extremity rehabilitation in patients with stroke in comparison to conventional physical therapy (CPT).

**Methods:**

In this study, a systematic search was conducted in various databases, including CINAHL, MEDLINE, PubMed, Embase, Cochrane Library, Scopus, Web of Science, and Physiotherapy Evidence Database (PEDro), as well as bibliographies of previous meta-analyses, to identify all randomized controlled trials that investigated the use of Lokomat^®^ devices in adult stroke patients. The study aimed to derive pooled estimates of standardized mean differences for six outcomes, namely, Fugl–Meyer Assessment lower-extremity subscale (FMA-LE), Berg Balance Scale (BBS), gait speed, functional ambulation category scale (FAC), timed up and go (TUG), and functional independence measure (FIM), through random effects meta-analyses.

**Results:**

The review analyzed 21 studies with a total of 709 participants and found that the use of Lokomat^®^ in stroke patients resulted in favorable outcomes for the recovery of balance as measured by the BBS (mean difference = 2.71, 95% CI 1.39 to 4.03; *p* < 0.0001). However, the FAC showed that Lokomat^®^ was less effective than the CPT group (mean difference = −0.28, 95% CI −0.45 to 0.11, *P* = 0.001). There were no significant differences in FMA-LE (mean difference = 1.27, 95% CI −0.88 to 3.42, *P* = 0.25), gait speed (mean difference = 0.02, 95% CI −0.03 to 0.07, *P* = 0.44), TUG (mean difference = −0.12, 95% CI −0.71 to 0.46, *P* = 0.68), or FIM (mean difference = 2.12, 95% CI −2.92 to 7.16, *P* = 0.41) between the Lokomat^®^ and CPT groups for stroke patients.

**Conclusion:**

Our results indicate that, with the exception of more notable improvements in balance, robot-assisted gait training utilizing the Lokomat^®^ was not superior to CPT based on the current literature. Considering its ability to reduce therapists' work intensity and burden, the way in which Lokomat^®^ is applied should be strengthened, or future randomized controlled trial studies should use more sensitive assessment criteria.

## Introduction

Stroke is a highly prevalent medical condition that often leads to permanent disability (1). Post-stroke impairment can have a significant impact on various aspects of physical function, including joint mobility and stability, muscular strength, tone, reflexes, muscle endurance, movement control, and gait pattern functions. These deficits can pose significant challenges to activities such as transferring, maintaining body posture, movement, balance, and walking (2). According to estimates, a considerable proportion of individuals who have suffered stroke, up to 65%, experience lower limb complications in the post-stroke phase (3). The ability to walk and achieve independence in activities of daily living (ADL) holds significant importance in various domains, including enhancing psychological wellbeing (4), mitigating the risk of cognitive decline (5), and promoting physical activity (6). The restoration of gait, both in terms of quantity and quality, is regarded as a primary objective (7).

Rehabilitation robotics is an emerging clinical intervention aimed at re-establishing functional movement of the limb by stimulating and restoring the nervous system that controls limb movement. This goal is achieved through multiple movements powered by robotic devices (8). Studies have shown that robot-assisted gait training triggers unique neurophysiological modulations (9). In addition, robot-assisted gait training enables patients to perform intense rehabilitation exercises in a safe manner while reducing the time and physical burden on physical therapists (10). Numerous empirical studies have supported the significant efficacy of robot-assisted interventions in the treatment of lower limb injuries after stroke (11). Stationary robot-assisted training can better improve the walking ability of subacute stroke survivors compared to traditional training methods. In addition, end-effector robots are superior to exoskeleton robots in improving step speed (12). However, some scholars have questioned the widespread use of body weight-supported running training (BWSTT) and robot-assisted gait training (RAST) in clinical practice through retrospective studies, arguing that physical therapists should remain cautious in adopting these strategies in the absence of sufficient evidence to support them (13).

Lokomat^®^ (Hocoma AG, Volketswil, Switzerland) is a globally utilized exoskeleton equipped with linear drives on the hip and knee joints designed to aid in locomotion on a treadmill by directing the participant's legs along a predetermined path (14). Prior research endeavors aimed at assessing the efficacy of Lokomat^®^ have predominantly amalgamated the lower-extremity robot, comprising both the end-effector and exoskeleton, and have scarcely scrutinized individual devices. Furthermore, the outcomes of such studies have primarily centered on gait kinematic parameters (15–17). Recent research has primarily examined the effects of Lokomat^®^ intervention on balance function in patients, with limited literature available to provide a comprehensive assessment of the restoration of lower limb function (18). It is essential to identify and employ the techniques that have been shown to produce the most significant results in order to lower the cost and improve the effectiveness of post-stroke rehabilitation. Hence, the aim of this study was to determine the effects of robot-assisted gait training with Lokomat^®^ in stroke patients on lower extremity function. In this study, FMA-LE was used as the primary outcome indicator, and BBS, gait speed, FAC, TUG, and FIM were used as secondary outcome indicators.

## Methods

**Figure 1:**
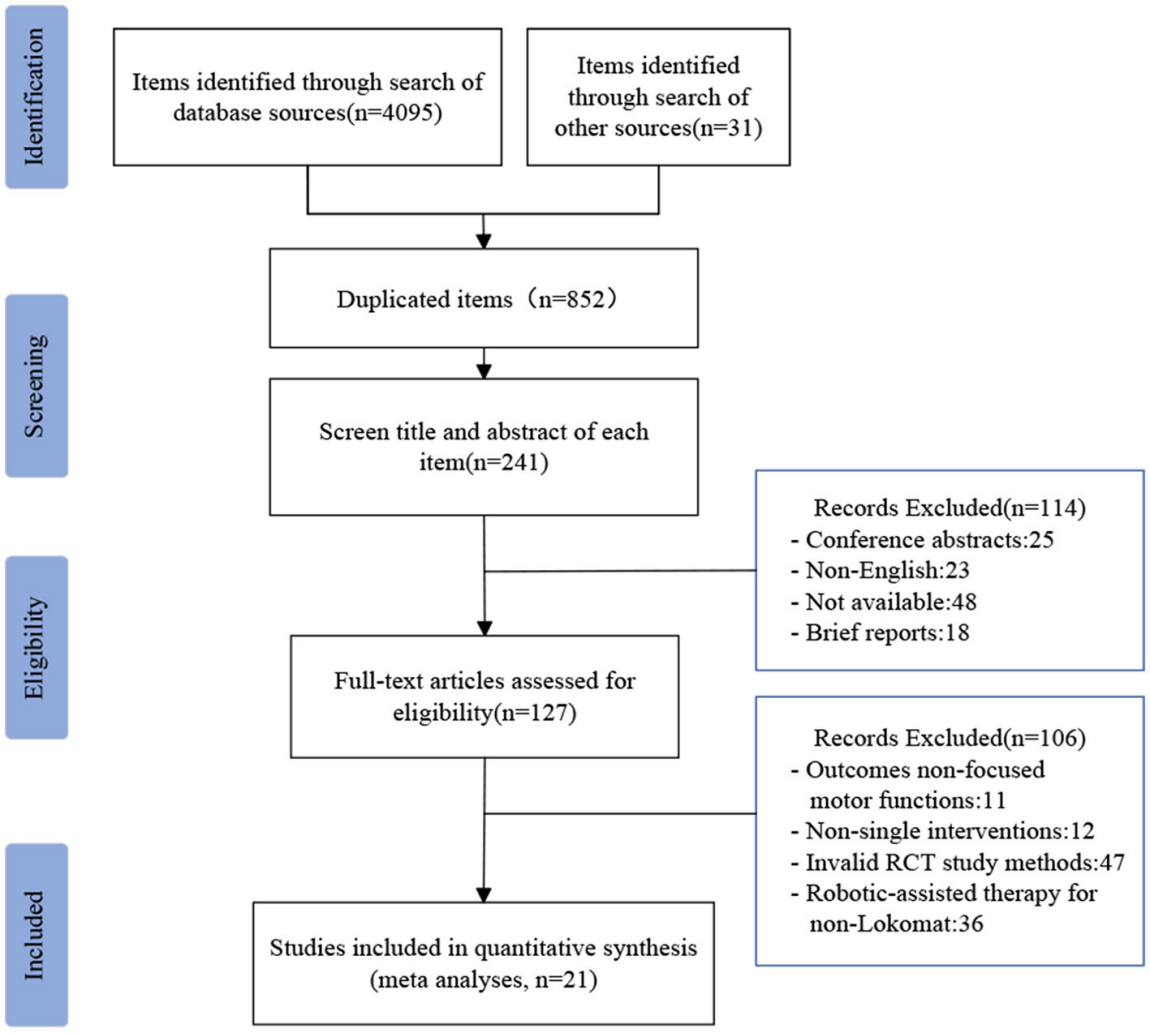
PRISMA flowchart showing the screening process.

In this study, we adhered to the guidelines set forth by the Preferred Reporting Items for Systematic Reviews and Meta-Analyses (PRISMA) in order to accurately report our findings from the systematic review (Figure 1) (19). The Cochrane handbook's published guidelines were strictly followed throughout the study (20). In addition, in this study, to ensure that the inclusion metrics were high-quality studies relevant to the study topic, we used predefined outcome measures to select studies. The protocol for this review is registered on PROSPERO (no. CRD42023438449).

### Search strategy

For relevant English-language literature, we searched the following electronic databases: CINAHL, MEDLINE, PubMed, Embase, Cochrane Library, Scopus, Web of Science, and Physiotherapy Evidence Database (PEDro). These databases define the search time to be from 1 January 2000 to 31 May 2023. The words stroke, cerebrovascular accident, CVA, Lokomat, robotic device, exoskeleton, robotic-assisted gait training, RAGT, gait, lower extremity function, and motion control were used in the literature search. Additionally, the PICOS framework (population, intervention, comparison, outcome, and study setting/design) was used in the design of this study.

### Study selection

Two authors (LW and GX) evaluated the title and summary of the studies that were found and then analyzed the complete reports of all studies that could be relevant based on predetermined criteria. Any discrepancies in the selection of studies were resolved through discussion with a third author (QW).

This study aimed to identify randomized controlled trials (RCTs) that compared the effectiveness of robotic-assisted gait training using Lokomat^®^ with CPT in improving lower limb function among stroke patients. The inclusion criteria for the RCTs were as follows: patients of both genders aged over 18 years with lower-extremity hemiparesis, outcome measures focused on motor function and limited walking ability after ischemic or hemorrhagic stroke, and sufficient cognitive abilities to comprehend the exercises involved in the interventions. Trials that utilized electromechanical devices other than Lokomat^®^ were excluded from the analysis.

### Data extraction

Two authors (LW and GX) conducted an independent extraction of information from each study included in the analysis. The extracted information included title, authors, country, year of publication, journal of publication, participants (number, mean age, and gender), study design, rehabilitative intervention details (frequency and duration of the sessions, Lokomat^®^ parameters such as weight support and speed), outcome measures, results, follow-up, attrition rates, and safety. Any discrepancies in the extracted data were resolved through discussion with another author (QW). In the context of reporting data on scale, the utilization of medians and interquartile ranges was accompanied by a reference to the methods proposed by Hozo et al. (21). These methods involve the application of basic inequalities and approximations to estimate the sample mean and standard deviation (SD), thereby facilitating subsequent analysis. In instances where it was deemed necessary, we initiated communication with the authors to obtain supplementary data.

### Risk of bias in included studies

Of the specific methods described for randomization, 20 out of 21 studies mentioned randomization, of which 12 specifically described randomization methods, 6 studies used computer-generated, 5 used random number table generation, and 1 used hidden envelope generation. In contrast, one study did not mention randomization. Allocation concealment to conceal enrollment identities, studies in 12 articles mentioned distribution concealment, with 7 detailing the use of opaque envelopes, 3 studies using a specific scale, and 2 only mentioning it without specifying how it was implemented. The remaining 9 did not report whether concealment was performed. Blinding of participants and staff: Due to the nature of the intervention, it was not possible to blind participants and research staff. Therefore, all studies were judged to be high risk. However, 15 of the included studies made reference to blinding the assessor. Incomplete outcome data: the field is unbiased.

### Assessment of the quality and methodology of literature inclusion

The evaluation of the methodological quality of the studies included in the research was carried out independently by two authors (LW and GX). The assessment was conducted using the PEDro scale (22), a validated and reliable tool for measuring methodological quality (23). The PEDro scale consists of 11 items, including eligibility criteria, random allocation, concealed allocation, similarity at baseline, subject blinding, therapist blinding, assessor blinding, follow-up of at least 85% for one key outcome, intention-to-treat analysis, between-group statistical comparison for one key outcome, and point and variability measures for one key outcome. The score of the first entry does not count toward the final overall score. If criteria are not specified, they are considered not met. The achieved criteria can be summed to obtain a score ranging from 0 (minimum) to 10 (maximum), indicating the overall methodological quality of the study ( ≤ 4 poor, 4–5 acceptable, 6–8 good, 9–10 excellent), (Table 1).

**Table 1:** Evaluation of the study's methodological quality using the PEDro criteria.

**Included studies**	**Eligibility criteria**	**Random allocation**	**Concealed allocation**	**Baseline comparability**	**Blind subject**	**Blind therapist**	**Blind assessor**	**Adequate follow-up**	**Intention-to-treat analysis**	**Betwee*n*-group comparisons**	**Point estimates and variability**	**Total score (0–10)**	**Methodological quality**
Bang et al. (25)	✓	✓	✓	✓	✓	✓	✓	✓	✓	✓	✓	7	Good
Belas et al. (26)	✓	✓	✓	✓	✓	✓	✓	✓	✓	✓	✓	4	Poor
Bergmann et al. (27)	✓	✓	✓	✓	✓	✓	✓	✓	✓	✓	✓	6	Good
Chang et al. (28)	✓	✓	✓	✓	✓	✓	✓	✓	✓	✓	✓	7	Good
Choi et al. (29)	✓	✓	✓	✓	✓	✓	✓	✓	✓	✓	✓	5	Acceptable
Ucar et al. (30)	✓	✓	✓	✓	✓	✓	✓	✓	✓	✓	✓	4	Poor
Han et al. (31)	✓	✓	✓	✓	✓	✓	✓	✓	✓	✓	✓	5	Acceptable
Hidler et al. (32)	✓	✓	✓	✓	✓	✓	✓	✓	✓	✓	✓	6	Good
Hornby et al. (33)	✓	✓	✓	✓	✓	✓	✓	✓	✓	✓	✓	5	Acceptable
Husemann et al. (34)	✓	✓	✓	✓	✓	✓	✓	✓	✓	✓	✓	7	Good
Kelley et al. (35)	✓	✓	✓	✓	✓	✓	✓	✓	✓	✓	✓	6	Good
Kim et al. (36)	✓	✓	✓	✓	✓	✓	✓	✓	✓	✓	✓	7	Good
Manuli et al. (37)	✓	✓	✓	✓	✓	✓	✓	✓	✓	✓	✓	6	Good
Mustafaoglu et al. (38)	✓	✓	✓	✓	✓	✓	✓	✓	✓	✓	✓	7	Good
Park et al. (39)	✓	✓	✓	✓	✓	✓	✓	✓	✓	✓	✓	5	Acceptable
Schwartz et al. (40)	✓	✓	✓	✓	✓	✓	✓	✓	✓	✓	✓	6	Good
Taveggia et al. (41)	✓	✓	✓	✓	✓	✓	✓	✓	✓	✓	✓	7	Good
Uivarosan et al. (42)	✓	✓	✓	✓	✓	✓	✓	✓	✓	✓	✓	3	Poor
Van Nunen et al. (43)	✓	✓	✓	✓	✓	✓	✓	✓	✓	✓	✓	5	Acceptable
Westlake et al. (44)	✓	✓	✓	✓	✓	✓	✓	✓	✓	✓	✓	6	Good
YUN et al. (45)	✓	✓	✓	✓	✓	✓	✓	✓	✓	✓	✓	6	Good

Eligibility criteria item does not contribute to total score.

### Data analysis

The analysis in this study was conducted using the Review Manager (RevMan) software (computer program, version 5.4, Copenhagen: The Nordic Cochrane Centre, The Cochrane Collaboration, 2020). In the case of each study that was incorporated and presented with continuous data, the between-group effect sizes were computed by contrasting the means between groups post-intervention. Furthermore, in instances where the outcome was evaluated by more than two RCTs, the pooled effects were determined using mean differences (MDs) with 95% confidence interval (CI) through random effects models that accounted for the differences in the use of instruments. The present study utilized pooled analyses, which were reported with 95% CI. The assessment of heterogeneity through the utilization of I^2^ statistics was interpreted in the following manner: when I^2^ = %, there is an absence of heterogeneity; when I^2^ >0% but < 25%, there is minimal heterogeneity; when I^2^ is ≥ 25% but < 50%, there is mild heterogeneity; when I^2^ ≥ 50% but < 75%, there is moderate heterogeneity; and when I^2^ ≥ 75%, there is strong heterogeneity (24).

## Results

### Literature search

A total of 4126 references were identified through various databases and additional sources. These included 200 references from CINAHL, 748 from PubMed, 408 from MEDLINE, 1024 from Embase, 74 from Cochrane Library, 869 from Scupos, 21 from PEDro, 751 from Web of Science, and 31 from other sources. Among these references, 3274 were duplicates, and 611 were excluded based on a review of their titles and abstracts. Further exclusions were made for 114 articles, which were deemed unsuitable due to reasons such as being conference abstracts, non-English publications, unavailability, or brief reports. Ultimately, a total of 127 articles were selected for full-text review. After careful examination, articles were excluded if they did not focus on motor function, did not involve single intervention factors, did not employ effective RCT study methods, or did not pertain to robotic treatment using the Lokomat^®^. As a result, 21 articles met the inclusion criteria and were included in the final analysis.

### Included studies

Table 2 displays the methodological characteristics and main results of the aforementioned studies.

**Table 2:** Methodological characteristics and main results of the included studies.

**Study (country)**	**Study design**	**Population (1 = RAGT; 2 = CPT)**	**Intervention (Lokomat^^®^^ characteristics)**	**Comparison**	**Outcomes**	**Follow-up**
Bang et al. (25) (Korea)	RCT	1. *n =* 9 (4F/5M; mean age 53.56 ± 3.94); 2. *n =* 9 (5F/4M; mean age 53.67 ± 2. 83)	Parameters: BWS (40%), speed (0.45 m/s); Frequency: 5 (1 h/session) for 4 weeks	Treadmill training without body support	BBS; Gait Speed; ABC	None
Belas et al. (26) (Brazil)	RCT	1. *n =* 7 (2F/5M; mean age 44.4 ± 12. 7); 2. *n =* 9 (2F/6M; mean age 56.4 ± 11. 8)	Parameters: BWS (50%), speed (1.5 km/h); Frequency: 3 (1 h/session/week) for 5 months	Therapist-assisted gait training + conventional treatment	BBS; TUG; FIMSARA	None
Bergmann et al. (27) (Germany)	RCT	1. *n =* 15 (5F/10M; mean age 72 ± 9); 2. *n =* 15 (8F/7M; mean age 71 ± 10)	Parameters: BWS (50%), speed (2 km/h); Frequency: 5 (1 h/session/week) for 2 weeks	Conventional treatment (consisted of active and dynamic exercises)	FAC; SCP; BLS; SVV	2-weeks/FAC
Chang et al. (28) (Korea)	RCT	1. *n =* 20 (7F/13M; mean age 55.5 ± 12. 0); 2. *n =* 17 (7F/10M; mean age 59.7 ± 12. 1)	Parameters: BWS (from 40% to 0%), guidance force (from 100% to 10%), speed (from 1.2 to 2.6km/h); Frequency: 10 (40 min/session/week) for 2 weeks	Conventional treatment (based on NDT developed Bobath)	FMA-LE; FAC; MI-L	None
Choi et al. (29) (Korea)	RCT	1. *n =* 6 (4F/2M; mean age 54.7 ± 12. 3); 2. *n =* 6 (3F/3M; mean age 61. 4 ± 9.7)	Parameters: BWS (50%); Frequency: 5 (1 h/session/week) for 6 weeks	Conventional physical therapy + gait training with treadmill + NDT	BBS; TUG	None
Ucar et al. (30) (Turkey)	RCT	1. *n =* 11 (0F/11M; mean age 56.2); 2. *n =* 11 (0F/11M; mean age 61. 5)	Parameters: BWS (50%), speed 1.5 km/h; Frequency: 5 (30 min/session/week) for 2 weeks	Conventional treatment (focused on gait)	TUG	8-weeks/TUG
Han et al. (31) (Korea)	RCT	1. *n =* 30 (13F/17M; mean age 67.89 ± 14.96); 2. *n =* 26 (11F/15M; mean age 63.2 ± 10.62)	Parameters: BWS (from 50% to 0%), guidance force (from 100% to 40%), speed (from 1.2 to 2.6km/h); Frequency: 5 (30 min/session/week) for 4 weeks	Conventional treatment (NDT)	FMA-LE; BBS; FAC	None
Hidler et al. (32) (United States)	RCT	1. *n =* 33 (12F/21M; mean age 59.5 ± 11. 3); 2. *n =* 30 (12F/18M; mean age 54.6 ± 9.4)	Parameters: BWS (40%), guidance force (100%), speed (1.5 km/h); Frequency: 3 (1.5 h/session/week) for 8~10 weeks	Conventional treatment (gait training)	FAC;	3-months/FAC
Hornby et al. (33) (United States)	RCT	1. *n =* 24 (9F/15M; mean age 57 ± 10); 2. *n =* 24 (9F/15M; mean age 57 ± 11)	Parameters: BWS (40%), speed (2~3 km/h); Frequency: 12 (30-min) sessions	Treadmill with BWS assisted by therapist	BBS; Gait Speed	None
Husemann et al. (34) (Germany)	RCT	1. *n =* 16 (5F/11M; mean age 60 ± 13); 2. *n =* 14 (4F/10M; mean age 57 ± 11)	Parameters: BWS (30%); Frequency: 20 (1 h) sessions	Conventional physiotherapy (gait training)	FAC; Gait Speed; MI-L; BI	None
Kelley et al. (35) (United States)	RCT	1. *n =* 11 (4F/7M; mean age 66.91 ± 8.50); 2. *n =* 19 (3F/6M; mean age 64.33 ± 10.91)	Parameters: BWS (40%), guidance force (100%), speed (0.42~0.89 m/s); Frequency: 5 (1 h/session/week) for 8 weeks	Conventional physiotherapy (endurance, velocity, safety, and gait deviations)	Gait Speed	3-months/ Gait Speed
Kim et al. (36) (Korea)	RCT	1. *n =* 10 (1F/9M; mean age 48.70 ± 7.01); 2. *n =* 9 (2F/7M; mean age 46.00 ± 15.64)	Parameters: BWS (from 80% to 50%), guidance force (from 100% to 20%), speed (1.0~3.0 km/h); Frequency: 5 (session/week) for 4 weeks	Conventional physiotherapy (static and dynamic balance)	FMA-LE; Gait Speed; FAC; TIS; SARA	None
Manuli et al. (37) (Italy)	RCT	1. *n =* 30 (26F/4M; mean age 40.1 ± 10.7); 2. *n =* 30 (16F/14M; mean age 43.1 ± 9.7)	Parameters: BWS (from 70% to 20%); Frequency: 5 (1 h/session/week) for 8 weeks	Conventional treatment (NDT)	FIM	None
Mustafaoglu et al. (38) (Turkey)	RCT	1. *n =* 15 (4F/11M; mean age 53.7 ± 11. 6); 2. *n =* 15 (4F/11M; mean age 52. 6 ± 14.7)	Parameters: BWS (40%), speed (1.2~2.6 km/h); Frequency: 2 times/week for 4 weeks	Conventional treatment (trunk stabilization, weight transfer)	BBS; TUG; RMI	None
Park et al. (39) (Turkey)	RCT	1. *n =* 12 (5F/7M; mean age 55.58 ± 10.42); 2. *n =* 16 (7F/9M; mean age 57.50 ± 9.90)	Parameters: BWS (30%), guidance force (100%), speed (1.5~2.0 km/h); Frequency: 3 (45 min/session/week) for 6 weeks	Gait training with treadmill	BBS; TUG; BI; FMA	None
Schwartz et al. (40) (Israel)	RCT	1. *n =* 37 (21F/16M; mean age 62 ± 85); 2. *n =* 30 (20F/10M; mean age 65 ± 75)	Parameters: BWS (from 50% to 10%); Frequency: 5 (30 min/session/week) for 6 weeks	Conventional physiotherapy (gait training)	Gait Speed; TUG; FIM	None
Taveggia et al. (41) (Italy)	RCT	1. *n =* 13 (6F/7M; mean age 71 ± 5); 2. *n =* 15 (5F/10M; mean age 73 ± 7)	Parameters: BWS (50%), speed (0.4 m/s); Frequency: 5 (session/week) for 5 weeks	Conventional physiotherapy (gait training)	Gait Speed; FIM	17-weeks/ Gait Speed; FIM
Uivarosan et al. (42) (Romania)	RCT	1. *n =* 18 (3F/15M; mean age 63.67 ± 6.63); 2. *n =* 30 (14F/16M; mean age 64.12 ± 7.25)	Parameters: speed (maximum that patients tolerate); Frequency: 14 (30 min/session/day) per 6 months	Recovery therapy (kinetotherapy + hydrokinetotherapy + masotherapy + electrotherapy et al.)	BI; FIM	None
Van Nunen et al. (43) (Netherlands)	RCT	1. *n =* 16 (6F/10M; mean age 50.0 ± 9.6); 2. *n =* 14 (8F/5M; mean age 56.0 ± 8.7)	Parameters: BWS (up to 10%), guidance force (up to 20%), speed (1.5 km/h, up to 2.5 km/h); Frequency: 2 h/week for 8 weeks	Overground assisted therapy	BBS; TUG; Gait Speed; FAC; RMI	36-weeks/ BBS; TUG; Gait Speed; FAC; RMI
Westlake et al. (44) (United States)	RCT	1. *n =* 8 (2F/6M; mean age 58.6 ± 16.9); 2. *n =* 8 (1F/7M; mean age 55.1 ± 13.6)	Parameters: BWS (35%), speed (2.5km/h); Frequency: 3 (30 min/session/week) for 4 weeks	Conventional physiotherapy (treated by skilled physical therapists/trainers)	FMA-LE; BBS; Gait Speed	None
Yun et al. (45) (Korea)	RCT	1. *n =* 18 (8F/10M; mean age 63.6 ± 8.3); 2. *n =* 18 (9F/9M; mean age 64.3 ± 8.4)	Parameters: BWS (50%), guidance force (100%), speed (1.1 km/h); Frequency: 5 (30 min/session/week) for 3 weeks	Conventional treatment (NDT)	FMA-LE; FMA; BBS;	4-weeks/ FMA-LE; FMA; BBS

RCT, randomized controlled trial; RAGT, robot-assisted gait training; CPT, conventional physical therapy; F, female; M, male; BWS, body-weight support; BBS, Berg Balance Scale; ABC, activities-specific balance confidence; TUG, Timed Up and Go; SARA, Scale for the Assessment and Rating of Ataxia; FIM, Functional Independence Measure; SCP, Scale for Contraversive Pushing; BLS, Burke Lateropulsion Scale; SVV, Subjective Visual Vertical; FAC, functional ambulation category scale; MI-L, leg score of Motricity Index; FMA-LE, Fugl-Meyer Assessment lower-extremity subscale; BI, Barthel Index; TIS, Trunk impairment scale; FES, Falls Efficacy Scale; RMI, Rivermead Mobility Index; FMA, Fugl–Meyer Assessment; NDT, neurodevelopmental technique.

### Design and participants

Stroke patients were recruited from stroke units or in-hospital rehabilitation centers in nine countries across Europe, Asia, and America. A total of 709 patients were included in the study, with 436 (59%) being male subjects. The average age of the participants was 58.43 years, ranging from 40 to 73 years. The average time from stroke onset to inclusion in the study was 13.7 months, with a range of 16.1 days to 10.5 years. Out of the 21 RCTs included in the analysis, seven studies conducted follow-up assessments at an average of 13 weeks after the completion of treatment, ranging from 2 to 36 weeks. The remaining 14 studies only reported post-treatment assessments.

### Characteristics of robot-assisted gait training with Lokomat^®^

The Lokomat system, manufactured by Hocoma in Volketswil, Switzerland, was the robotic device utilized in this research. The Lokomat^®^ device is employed alongside a body weight support (BWS) system, which assists in offsetting a portion of the individual's weight.

All 20 studies provided specific experimental parameters, except for one study that did not include machine setup parameters (42). These parameters included body support weight, guiding force, step speed, and treatment frequency time. The majority of studies reported body support weights ranging from 30% to 50%, with some studies noting that 30% was the most commonly used weight (46). Guidance force varied from 20% to 100%, and the initial training pace was approximately 1 km/h, gradually increasing to 3 km/h over the training period. The minimum number of training sessions in a treatment cycle was 8, the maximum was 60, and the median was 20 (26, 38, 41, 43). For a single session, the minimum duration was 30 min, the maximum was 2 h, and the median was 45 min (30, 31, 33, 40, 42, 44, 45).

The details of the Lokomat^®^ setup parameters and treatment frequency can be found in Table 2.

### Characteristics of CPT

In the studies analyzed, the CPT group received treatment of similar duration and frequency as the experimental group. The included studies encompassed four that utilized neurodevelopmental therapy (NDT)-based rehabilitation methods, four that incorporated treadmill training, and the remaining CPT interventions consisted of gait training, dynamic and static exercises, trunk control, and balance training (25, 28, 29, 31, 33, 37, 39, 45).

### Evaluation of the study's methodological quality with the PEDro criteria

Based on the evaluation using the PEDro scale, it was determined that out of the total number of studies assessed, 3 were classified as poor (26, 30, 42), 5 as acceptable (29, 31, 33, 39, 43), and 13 as good (25, 27, 28, 32, 34–38, 40, 41, 44, 45), indicating a generally high quality of literature. It is worth noting that only two studies reviewed did not make mention of randomization (26, 42), while eight studies reported allocation concealment (25, 27, 28, 33, 34, 36, 44, 45). Furthermore, 14 studies were found to have employed blinding techniques to the assessor (25–28, 30, 31, 34–41), with three of them extending this blinding the statistical analysts (25, 37, 41). Due to the inherent characteristics of the intervention, it was not feasible to implement blinding for both participants and researchers. Consequently, none of the studies were scored in either of these two aspects.

### Adverse events

Only one study mentioned the occurrence of an adverse event, specifically: 12 skin changes (redness or breakage of the skin due to pressure or friction on the shoulder strap or cuff) in 5 Lokomat^®^ participants (35).

### Meta-analysis results

#### Primary outcome

##### FMA-LE

**Figure 2:**
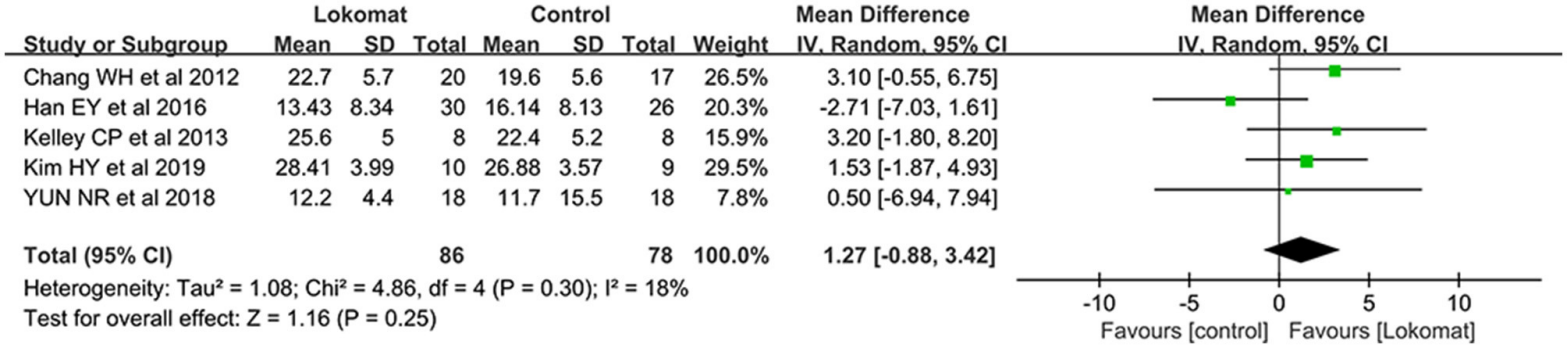
Forest plot: comparison of the effect of robot-assisted gait training with the Lokomat^®^ and CPT on FMA-LE at post-treatment.

A total of five studies were included in the assessment of the Functional Mobility Assessment of the Lower Extremities (FMA-LE), involving a total of 164 patients. The studies exhibited minimal heterogeneity (I^2^ = 18%, *P* = 0.30). A meta-analysis was conducted using a random effects model, which revealed no significant difference in the combined effect [MD = 1.27, *P* = 0.25, 95% (CI −0.88, 3.42)]. This suggests that there is no statistically significant distinction between Lokomat^®^ robot-assisted gait training and CPT in terms of their impact on lower limb functional training (Figure 2).

#### Secondary outcome

##### BBS

**Figure 3:**
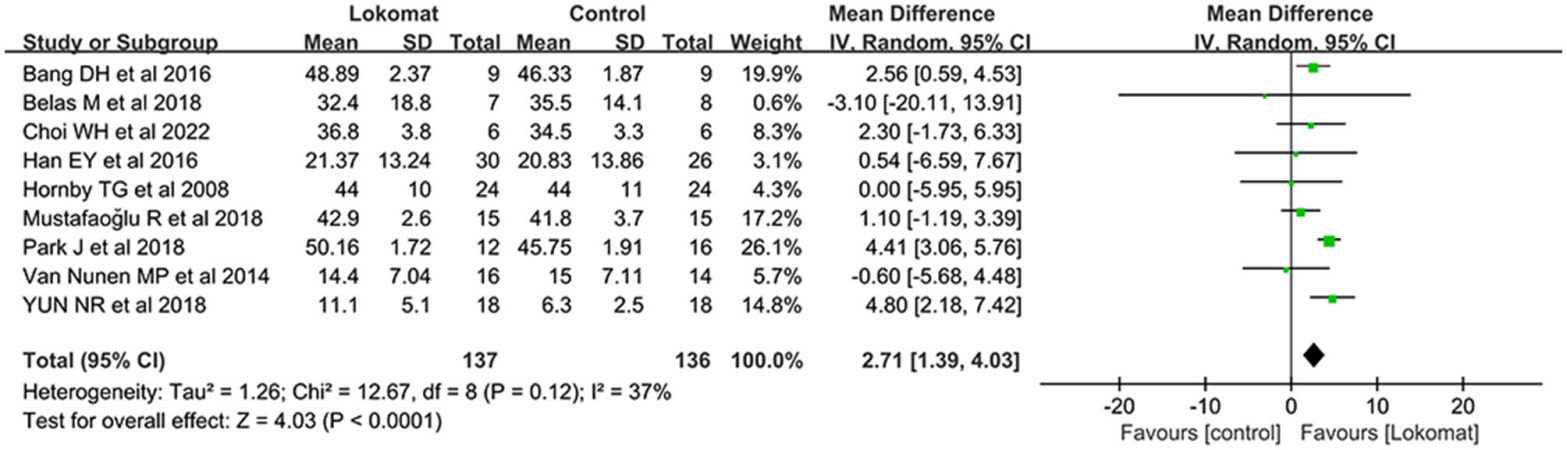
Forest plot: comparison of the effect of robot-assisted gait training with the Lokomat^®^ and CPT on BBS at post-treatment.

A comprehensive analysis was conducted on a total of nine studies involving 273 patients to evaluate the effectiveness of Lokomat^®^ robot-assisted gait training compared to CPT in improving balance function. The study groups exhibited mild heterogeneity (I^2^ = 37%, *P* = 0.12), and a random-effects model was employed for the meta-analysis. The results revealed a significant difference in the combined effect [MD = 2.71, *P* < 0.01, 95% CI (1.39, 4.03)], indicating that Lokomat^®^ robot-assisted gait training was more efficacious in enhancing balance function when compared to CPT (See Figure 3).

##### Gait speed

**Figure 4:**
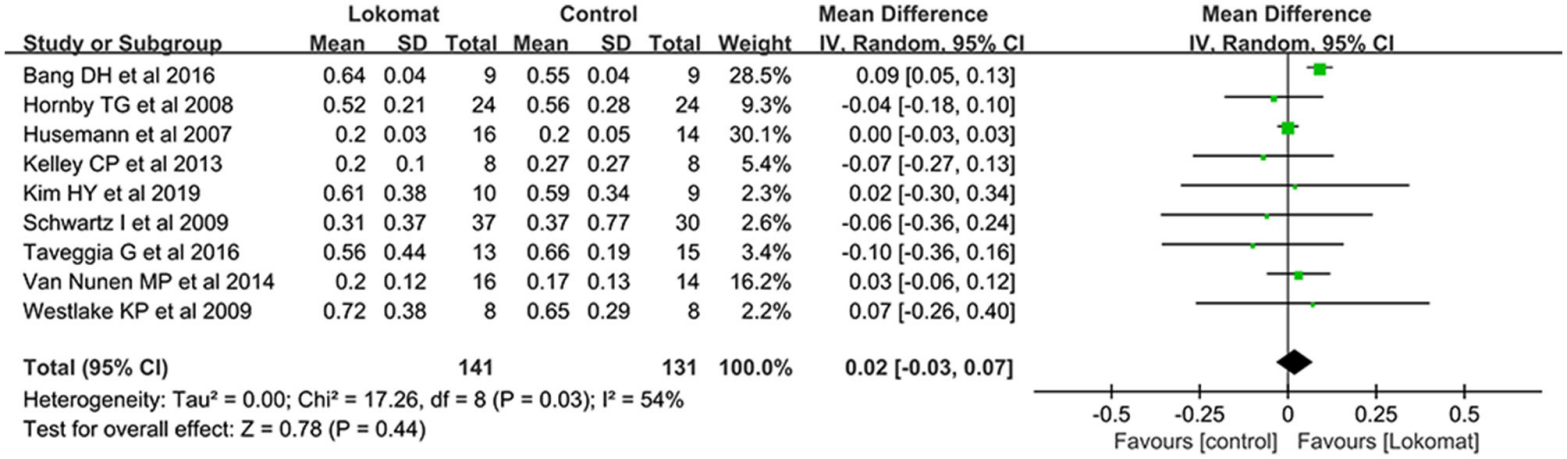
Forest plot: comparison of the effect of robot-assisted gait training with the Lokomat^®^ and CPT on gait speed at post-treatment.

Gait speed was chosen as an outcome measure in a total of nine studies involving 272 patients. The study groups exhibited moderate heterogeneity (I2 = 54%, *P* = 0.03), and a meta-analysis was conducted using a random-effects model. The results showed no significant difference in the combined effect [MD = 0.02, *P* = 0.44, 95% CI (−0.03, 0.07)], indicating that Lokomat^®^ robot-assisted gait training does not offer a significant advantage over CPT in terms of improving walking speed (Figure 4).

##### FAC

**Figure 5:**
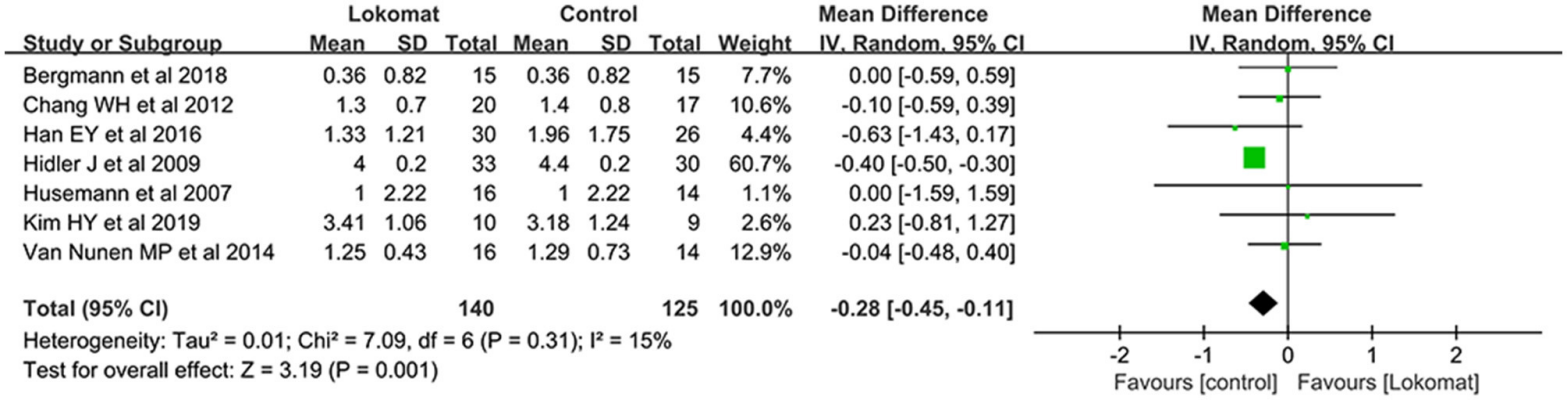
Forest plot: comparison of the effect of robot-assisted gait training with the Lokomat^®^ and CPT on FAC at post-treatment.

A comprehensive analysis was conducted on a total of seven studies involving 275 patients to evaluate the effectiveness of Lokomat^®^ robotic-assisted gait training compared to CPT in improving functional walking. The study groups exhibited a slight degree of heterogeneity (I^2^ = 15%, *P* = 0.31). A meta-analysis was performed using a random-effects model, revealing a significant difference in the combined effect [MD = −0.28, *P* < 0.01, 95% CI (−0.45, −0.11)]. This indicates that Lokomat^®^ robotic-assisted gait training did not demonstrate superiority over CPT in enhancing functional walking (Figure 5).

##### TUG

**Figure 6:**
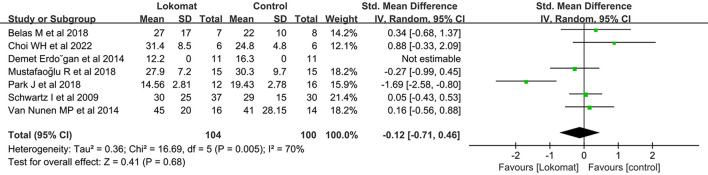
Forest plot: comparison of the effect of robot-assisted gait training with the Lokomat^®^ and CPT on TUG at post-treatment.

A systematic review was conducted to assess the effectiveness of Lokomat^®^ robot-assisted gait training compared to CPT in improving the time to complete the TUG. A total of seven studies involving 204 patients were included in the analysis. The study groups exhibited moderate heterogeneity (I^2^ = 70%, *P* < 0.01). A meta-analysis was performed using a random-effects model, which revealed no significant difference in the combined effect [MD = −0.12, *P* = 0.68, 95% CI (−0.71, 0.46)]. These findings suggest that Lokomat^®^ robot-assisted gait training is not significantly superior to CPT in terms of enhancing TUG performance (Figure 6).

##### FIM

**Figure 7:**
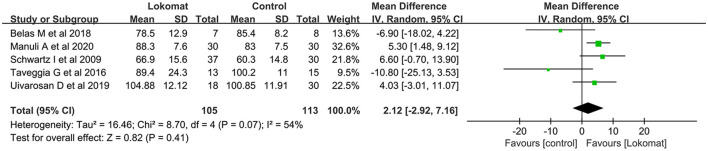
Forest plot: comparison of the effect of robot-assisted gait training with the Lokomat^®^ and CPT on FIM at post-treatment.

A selection of five studies involving a total of 218 patients was utilized to assess the Functional Independence Measure (FIM). The study groups exhibited a moderate level of heterogeneity (I2 = 54%, *P* = 0.07). A meta-analysis was conducted using a random-effects model, which revealed no significant difference in the combined effect (MD = 2.12, *P* = 0.41, 95% CI [−2.92, 7.16]). This suggests that there is no substantial disparity in the improvement of functional independence between Lokomat^®^ robot-assisted gait training and CPT (Figure 7).

## Discussion

The existing studies lack a comprehensive evaluation of the effectiveness of robot-assisted gait training using the Lokomat^®^ compared to CPT for lower extremity rehabilitation. Therefore, the purpose of this systematic review is to fill this gap. The literature examined in this review demonstrates a commendable level of quality, with the majority of studies being deemed acceptable and good. As a result, our conclusions can be considered informative.

In a meta-analysis conducted by Baronchelli et al. (18), the authors examined the recovery of balance function in stroke patients using three different balance scales in comparison to traditional physical therapy. The findings indicated that Lokomat^®^ robot-assisted walking training was more effective in improving the TUG test and the Rivermead Mobility Index, while the results for BBS were inconclusive. The inconsistency in the BBS results can be attributed to the fact that Baronchelli et al. calculated the difference between pre- and post-treatment outcome measures, whereas our study combined the results after treatment, leading to inconsistent findings. We argue that our approach aligns with the guidelines outlined in the Cochrane Handbook for Systematic Reviews of Interventions. In terms of motor function, our analysis of the functional ambulation category (FAC) yielded consistent results with a study conducted by Calafiore et al. (16), showing no significant difference between the two interventions. It is worth noting that the wide range of grading in the FAC scale may make it challenging to detect treatment differences within a short intervention period. Additionally, other studies have reported that the robot did not outperform traditional therapy in areas such as daily life function and walking speed (47, 48). The early design of the robot also restricted trunk and pelvic movements, which negatively affected pelvic movements during gait training (32). This limitation may explain the lack of observed improvement in gait in some studies.

The findings of the present study indicate that the development of robotic devices has aimed to alleviate the physical strain associated with repetitive manual-assistance tasks and enhance the quality of gait performance and to provide assistance to therapists and patients throughout different phases of neurorehabilitation. While there is scientific and clinical evidence supporting the efficacy, safety, and tolerability of gait training with robotic devices, there is a scarcity of documentation regarding their comparative advantages over conventional therapies. Currently, there is a focus on enhancing the assisted gait patterns by utilizing sensors and control algorithms in order to improve their quality (49). Recent studies have demonstrated that the utilization of robot-assisted treadmill training led to a more balanced distribution of muscle activity in individuals with paresis as opposed to the conventional treatment methods (50). Another aspect of interest is that Lokomat^®^ robot-assisted walking training can reduce the burden on the therapist when the training is more intense and longer in duration (51). Although no such data were obtained in this study, this is an important reason for us to recommend the promotion and use of Lokomat^®^ even after drawing this conclusion. With the exception of Kelley et al. (35), who documented study-related adverse events (AEs) such as skin redness or breakage caused by pressure or friction from the straps or cuffs, no other studies reported any adverse events. Consequently, the utilization of the Lokomat^®^ robot is generally considered to be safe.

It is conceivable that the diverse outcome measures employed in the literature reviewed may obscure the potential benefits derived from the Lokomat^®^. In essence, the outcome measures utilized in the articles may not accurately reflect the true effects of the treatment. Consequently, there is a necessity for more refined and objective scales to comprehensively assess the clinical outcomes, enabling a more accurate understanding of the treatment's efficacy. Furthermore, the utilization of the Lokomat^®^ in gait training heavily relies on the therapist's personal experience and familiarity with the device. While efforts are continuously made to refine standard rehabilitation protocols, it is evident that the use of the robot necessitates individualized treatment. It is imperative to perceive it as a tool rather than a ready-made solution, thus necessitating further investigation into treatment duration, support weights, walking parameters, and other relevant factors to optimize its utility for physiotherapists.

Potential discrepancies in the findings of this study may have arisen from various factors, including the quality and language of the literature incorporated. First, with regard to the quality of the literature, it is worth noting that while all the included trials were RCTs, the adequacy of blinding and randomization procedures in individual studies was not consistently well executed. Second, the restriction to English language literature may have resulted in the exclusion of relevant studies published in other languages. Unavailability due to non-publication or non-appearance in publicly available databases introduces a certain amount of uncertainty when analyzing the results. This may result in our results not being comprehensive enough to capture the true effect across the field. Therefore, a certain amount of caution is introduced when interpreting our results and presenting conclusions. In addition, we recommend that future studies consider reporting their findings more comprehensively and make them as accessible as possible so that further meta-analysis can better reveal the true picture of the effect of the Lokomat^®^ on motor function rehabilitation after stroke. Although the assessment of literature quality adhered to basic criteria across the board, it is important to acknowledge the presence of heterogeneity in the results, indicating a potential lack of reliability.

In general, the system is easily configured, minimizes the requirement for physical therapy labor, and aligns with human expectations of robotic assistance. Nevertheless, the findings of the present investigation indicate suboptimal clinical outcomes in lower extremity rehabilitation, particularly in relation to exercise. Consequently, additional improvements in the implementation and assessment approaches are necessary.

## Author contributions

LW: Formal analysis, Methodology, Software, Writing—original draft, Writing—review & editing. GX: Data curation, Investigation, Writing—review & editing. QW: Data curation, Investigation, Writing—review & editing.
